# The Temporal Distribution of Cyclic Imines in Shellfish in the Bays of Fangar and Alfacs, Northwestern Mediterranean Region

**DOI:** 10.3390/toxins16010010

**Published:** 2023-12-23

**Authors:** Lourdes Barreiro-Crespo, Margarita Fernández-Tejedor, Jorge Diogène, Maria Rambla-Alegre

**Affiliations:** 1 Institute of Agrifood Research and Technology (IRTA), Ctra. Poble Nou km.5, 45350 La Ràpita, Spain; lourdes.barreiro@irta.cat (L.B.-C.); margarita.fernandez@irta.cat (M.F.-T.); jorge.diogene@irta.cat (J.D.); 2 Department of Analytical Chemistry and Organic Chemistry, Universitat Rovira i Virgili, Marcel·lí Domingo s/n, 43002 Tarragona, Spain

**Keywords:** marine biotoxins, cyclic imines (CIs), spirolides (SPXs), gymnodimines (GYMs), pinnatoxins (PnTXs), shellfish, bivalves, mass spectrometry, human health, risk assessment

## Abstract

Spirolides (SPXs), gymnodimines (GYMs), and pinnatoxins (PnTXs) have been detected in shellfish from the northwestern Mediterranean coast of Spain. Several samples of bivalves were collected from Fangar Bay and Alfacs Bay in Catalonia over a period of over 7 years (from 2015 to 2021). Shellfish samples were analyzed for cyclic imines (CIs) on an LC1200 Agilent and 3200 QTrap triple–quadrupole mass spectrometer. In shellfish, SPX-1 was detected in two cases (of 26.5 µg/kg and 34 µg/kg), and GYM-A was only detected in trace levels in thirteen samples. Pinnatoxin G (PnTX-G) was detected in 44.6% of the samples, with its concentrations ranging from 2 µg/kg to 38.4 µg/kg. Statistical analyses revealed that seawater temperature influenced the presence or absence of these toxins. PnTX-G showed an extremely significant presence/temperature relationship in both bays in comparison to SPX-1 and GYM-A. The prevalence of these toxins in different bivalve mollusks was evaluated. A seasonal pattern was observed, in which the maximum concentrations were found in the winter months for SPX-1 and GYM-A but in the summer months for PnTX-G. The obtained results indicate that it is unlikely that CIs in the studied area pose a potential health risk through the consumption of a seafood diet. However, further toxicological information about CIs is necessary in order to perform a conclusive risk assessment.

## 1. Introduction

Marine toxins include a wide range of natural substances that have extremely diverse mechanisms of action, biological activities, and molecular structures. They have molecular masses ranging from 300 Da to 3000 Da, and they have been classified in various ways according to their mechanisms of action, their structural similarity in terms of their degree of solubility in organic solutions (e.g., lipophilic, hydrophilic, and amphiphilic), and the symptoms they induce in humans after poisoning (e.g., paralytic shellfish poisoning (PSP), diarrheic shellfish poisoning (DSP), amnesic shellfish poisoning (ASP), neurotoxic shellfish poisoning (NSP), and ciguateric fish poisoning (CFP)). Lipophilic toxins include several groups, namely okadaic acid (OA), pectenotoxins (PTXs), yessotoxins (YTXs), and azaspiracids (AZAs), with OA and its derivatives dinophysistoxins (DTXs) considered the best known and most frequent worldwide.

Catalonia (Spain) ranks second in Spain in terms of the most robust mussel aquaculture. In 2022, 2905.2 tons of mussels, 397.31 tons of oysters, and 6.3 tons of clams were produced (IDESCAT, Aquaculture Production by Species from the Ministry of Climate Action, Food, and Rural Agenda of Catalonia, 2022). Notably, 90% of northwestern (NW) Mediterranean farm areas are situated in Alfacs Bay and Fangar Bay within the Ebro Delta. Both bays have shallow depths, freshwater inputs, and relatively calm waters, and provide shelter from coastal circulation. Fangar Bay (12 km^2^) is located in the northern part of the Ebro Delta, with a maximum depth of approximately 4 m. Alfacs Bay is larger than Fangar Bay; it is located in the southern part of the Ebro River Delta, and it has a surface area of 49 km^2^ and a maximum depth of about 6 m. The water renewal time is 20–70 days for Alfacs Bay [1] and 2–5 days for Fangar Bay [2].

In Catalonia, there is little information about the emerging lipophilic toxins present in bivalve mollusks, as well as the microalgal sources associated with their production and their temporal variability. Additionally, due to the present discussion on the potential hazards that CIs may present, this group of toxins deserves higher attention.

CIs include spirolides (SPXs), gymnodimines (GYMs), and pinnatoxins (PnTXs), with important structural differences [3,4,5]. All cyclic imines (CIs) have a structure formed by a macrocyclic skeleton of 12 to 27 carbon atoms, with a cyclic imine and a spirocentric ring at their ends [6], which seems to be the cause of their toxicity [7] (Figure S1).

The toxicity of these compounds in humans has not yet been demonstrated, but a high level of neurotoxicity was observed after their administration in mice via intraperitoneal injection. As they induce rapid death, CIs are known as “fast-acting” toxins [8]. All of them are powerful neurotoxic molecules, whose mechanism of action is related to the inhibition of the muscarinic receptors (mAChRs) and nicotinic receptors (nAChRs) of acetylcholine [9]. These receptors are the mediators of neurotransmission in the central and peripheral systems.

The concentration of CIs in bivalve mollusks is not regulated in Europe nor in other parts of the world [3]. Due to the lack of quantitative data on the acute oral toxicity of this group of emerging marine toxins [3,10], the EFSA has not been able to establish a reference dose. In a previously published report [3], it was mentioned that the EU Community Reference Laboratory on Marine Biotoxins Working Group on Toxicology recommends not exceeding 400 µg/kg in meat for SPX-1 [11,12]. Additionally, the National Food Safety Agency (ANSES) published an opinion request where a PnTX-G level of 23 µg/kg was recommended [13].

SPXs are polyether compounds that contain a spiro group attached to tricyclic ethers and are mainly produced by *Alexandrium ostenfeldii,* a thecate dinoflagellate that was first isolated in Iceland and described by Paulsen in 1904 [14,15,16]. They were identified, for the first time, in mussels off the coast of Nova Scotia (Canada, 1995) [17], although they are currently found in countries such as Spain, France, Italy, Scotland, Norway, Denmark, Mozambique, Chile, and Asia–Pacific countries [18,19,20,21,22,23,24,25]. The reference compound for this group is 13-desmethyl SPX-C (SPX-1) [26], which, together with SPX-C and 20-methyl SPX-G, is the most toxic compound [8].

GYMs were first detected in oysters of the species *Tiostrea chilensis* in New Zealand in the early 1990s [8]. These compounds are produced by the dinoflagellates *Karenia selliformis* and the species *A. ostenfeldii* [19], which suggests the existence of common biosynthetic pathways between *Karenia selliformis* and species of the genus *Alexandrium* [8]. The reference compound of this group is GYM-A [26]. Currently, GYMs have been detected on the coasts of Tunisia [27], Africa, Europe, and North America [28]. The occurrence of these toxins represents several concerns, as they have been found to accumulate in bivalve mollusks such as mussels (*M. galloprovincialis*), oysters (*T. chilensis*), scallops (*Pecten novaezelandiae*), and clams (*Ruditapes decussates*) [8].

PnTXs were first identified from extracts of the digestive gland of a bivalve mollusk from the South China Sea called *Pinna attenuata* [8]. The first analogue of PnTX-A was isolated from the shellfish *Pinna muricata*, harvested on the Japanese island of Okinawa (1995) [29,30], and about a decade ago, a dinoflagellate producing PnTX-E and F was isolated, but not identified, from shallow water samples from Rangaunu Harbor in northern New Zealand [31]. This group of toxins is produced by the dinoflagellate *Vulcanodinium rugosum* [9] and includes seven analogues whose chemical structure is very similar to that of the SPXs [3], where pinnatoxin-G (PnTX-G) is the reference compound [26]. These compounds are currently appearing in oysters (*T. chilensis*), mussels (*M. galloprovincialis*), razor clams *(Ensis magnus)*, and clams *(Ruditapes decussates)* from many countries such as Mozambique, Chile, Japan, China, South Australia, New Zealand, Canada, Norway, France, and Spain [8,19,21,22,23,24,25].

The appearance of emerging toxins is one of the major problems that concerns the EU. Monitoring the concentrations of marine toxins in water and shellfish (especially before harvesting) through efficient analytical methods [32] has become a priority objective for the EFSA [28]. Since 2015, the reference method for the analysis of lipophilic toxins in the EU is liquid chromatography coupled to tandem mass spectrometry (LC-MS/MS) (Regulation (EU) No. 15/2011 of the Commission, 2011), which was developed as an alternative to mouse bioassays (MBAs). Emerging toxins, such as Cis, lack official detection methods by the EU but are often included in the monitoring of lipophilic toxins via LC-MS/MS, since the same analysis that is carried out for legislated marine toxins can retrieve information on emerging toxins from all study areas. 

In most cases, CI concentrations found in the mollusks are very low (0.1–12 µg/kg PnTXs; 26–66 µg/kg SPX-1) in European commercial seafood [19]. In Galician waters (Spain), PnTXs were detected in shellfish from the Atlantic and Cantabrian coasts of Spain at low levels ranging between 0.36 µg/kg and 14.98 µg/kg of PnTXs [33], from 1.3 µg/kg to 23.9 µg/kg of GYM-A [34], and from 1.2 µg/kg to 6.9 µg/kg of SPX-1 [30], and being in the 75% of the results below 10 µg/kg [35]. In Norway, their levels were generally low, and CI concentrations reached 115 µg/kg of PnTX-G and 226 µg/kg of SPX-1 [36]. In Canada, levels of PnTX-G were below 80 μg/kg. Meanwhile, maximum concentrations of PnTXs have been detected in New Zealand, Norway, and the French Mediterranean, with their maxima below 200 µg/kg, 115 µg/kg, and 1244 µg/kg, respectively [29,36,37]. GYMs have been also found in Tunisia [27], France [38], and Italy [39], reaching moderate concentrations (maximum level of 12.1 µg/kg). 

Although hydrophilic toxins and amphiphilic toxins, which possess both hydrophilic and lipophilic properties, are the most dangerous toxins, lipophilic toxins are the most frequent in the northwestern Mediterranean region (Catalonia, Spain). In 2015, the presence of CIs on the Catalan coasts was detected [26], and since then, they have become a subject of great interest in this area, although they are not regulated. In this study, 1007 bivalve samples from January 2015 to April 2021 with a high-sensitivity mass spectrometer have been examined to determine whether CIs are present in this area, along with their trends depending on the season, the mollusk species, temperature, and area.

## 2. Results

**Figure 1:**
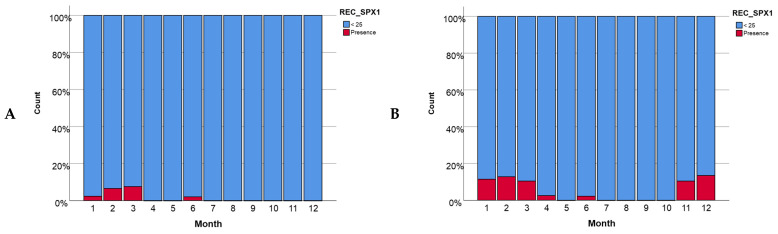
The presence of SPX-1 per month (**A**) in Alfacs Bay and (**B**) in Fangar Bay.

From January 2015 to April 2021, 1007 samples were analyzed, and SPX-1 was present in 36 of these samples (3.6%). Two samples presented levels between 26.5 µg/kg and 34 µg/kg, and all the other samples showed traces of SPX-1 (below 25 µg/kg). These results are consistent with other studies on Galician waters, in which SPX-1 levels ranged from 1.3 µg/kg to 23.93 µg/kg [34]. The presence/absence of SPX-1 related with the water temperature, mollusk species, and temporal distribution were evaluated through conducting statistical analyses. For water temperature, a Nagelkerke R^2^ value of 0.063 was observed for Alfacs Bay (Figure S2A), while for Fangar Bay (Figure S2B), a Nagelkerke R^2^ value of 0.101 was obtained. These values indicate that the effect of temperature was significant, being more important in Fangar Bay. In Alfacs Bay, the presence of SPX-1 was concentrated at intermediate temperatures around 12 °C (corresponding to 1 on a logarithmic scale, °C), while in Fangar Bay, the presence of this toxin began to be detected at temperatures between 5 °C and 10 °C (corresponding to 0.60 and 0.80 on a logarithmic scale, °C). The presence of SPX-1 in the different mollusks species was much higher in Fangar Bay (Figure S3B) than in Alfacs Bay (Figure S3A). In Alfacs Bay (Figure S3A), there was a greater tendency for the accumulation of SPX-1 in mussels (*M. galloprovincialis*) (5%), followed by oysters (*C. gigas*) (3%) and razor clams (*Ensis* sp.) (1%). In the case of Fangar Bay, the presence of this toxin was concentrated in oysters (10%), which are the main species produced in this bay, while razor clams are not harvested. Throughout the year, a variation in the presence of SPX-1 in both bays was observed (Figure 1), although certain similarities in some months were found. For both Alfacs Bay (Figure 1A) and Fangar Bay (Figure 1B), a greater presence of SPX-1 was observed during the winter and spring months. In Alfacs Bay (Figure 1A), the presence of this toxin was centered during the first quarter of the year and in June, while in Fangar Bay (Figure 1B), it was present during this same period and also in November and December. Thus, a seasonal pattern, with maximum levels of SPX-1 observed during the winter season (with temperatures between 5 °C and 15 °C), was observed. The presence of *A. ostenfeldii* was detected in two water samples from Alfacs Bay (February and December of 2020). 

**Figure 2:**
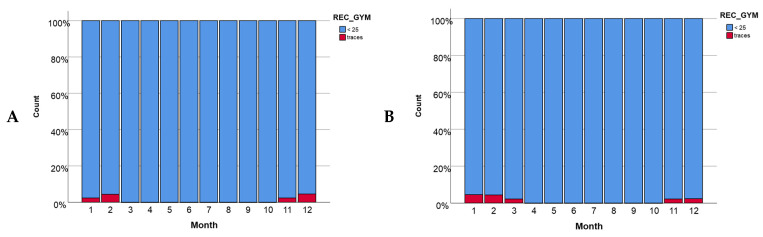
The presence of GYM-A per month (**A**) in Alfacs Bay and (**B**) in Fangar Bay.

For the same time period (from January 2015 to April 2021), traces of GYM-A were detected in 13 samples (1.3%). The recorded levels were all below 25 µg/kg. For GYM-A, the effect of the temperature for both bays was much smoother (Figure S4) than in the case previously shown for the levels of SPX-1 (Figure S2). The Nagelkerke R^2^ values of 0.068 for Alfacs Bay were obtained (Figure S4A), while for Fangar Bay (Figure S4B), the Nagelkerke R^2^ value was 0.073. These values indicate that the effect of temperature in this case was slightly significant, although it was still more important in Fangar Bay than in Alfacs Bay. In Alfacs Bay (Figure S4A), the presence of GYM-A was concentrated at intermediate temperatures of 12 °C (0.8 on a logarithmic scale, °C) while in Fangar Bay (Figure S4B), the presence of this toxin began to be detected at slightly lower temperatures of around 10 °C (0.7 on a logarithmic scale, °C). Regarding the bivalve mollusks species that were analyzed, significant differences between the species of each bay were observed. The highest concentration of GYM-A was found in the oysters (5% in Alfacs Bay (Figure S5A) and 2.5% in Fangar Bay (Figure S5B)). Lamas et al. [34] suggested that on the whole northern coast of Spain, from April 2017 to December 2019, the prevalence was not the same across all species, with clams being less prevalent than mussels, oysters, and razor clams. Similarly, the presence of GYM-A changes throughout the year, with a greater presence of this toxin in the winter months for both bays. In Alfacs Bay (Figure 2A), the presence of GYM-A was found to be slightly higher in the months of December and February and lower in the months of November and January. In Fangar Bay (Figure 2B), a similar trend occurred, with the winter season being the one where the greatest presence was recorded. *A. ostenfeldii* or *Karenia* species could be involved, since both are known to be present in this area. *A. ostenfeldii* was detected in two water samples from Alfacs Bay. *Karenia* species were frequently detected and reached high abundance (2 × 10^5^ cells/L) in November 2019. In half of these cases (54%), GYM-A and SPX-1 were concurrently detected, suggesting that *A. ostenfeldii* could be the responsible producer species. The existence of cases (46%) in which only GYM-A was detected alone also suggests that a *Karenia* species could be involved.

During the same period of time, from January 2015 to April 2021, PnTX-G presented an extremely significant presence/temperature relationship in both bays (Figure S6). In Alfacs Bay (Figure S6A), a Nagelkerke R^2^ value of 0.182 was observed, as well as in Fangar Bay (Figure S6B), where a Nagelkerke R^2^ value of 0.391 was presented. These high values of R^2^ indicate that the effect of temperature was significant in both bays. In Alfacs Bay (Figure S6A), the greatest presence of PnTX-G was found at temperatures from 16 °C to 25 °C (1.2–1.4 on a logarithmic scale, °C), while in Fangar Bay (Figure S6B), the presence of PnTX-G was reduced at temperatures below 18 °C – 20 °C (1.25–1.3 on a logarithmic scale, °C). The statistical study on the presence of PnTX-G by species in the two bays indicates the existence of significant differences at the levels of the species and bays. In Alfacs Bay (Figure S7A), the greatest presence of this toxin was concentrated in mussels (around 70%), followed by razor clams (68%) and oysters (2%). Similarly, PnTX-G was concentrated in mussels (around 50%) in Fangar Bay (Figure S7B), although not to such a high percentage as in Alfacs Bay. Farmed mussels that were described by Lamas et al. [33] seem to accumulate less PnTX-G than wild ones, concluding that there were significant differences in PnTX-G concentration between species and between habitats. 

**Figure 3:**
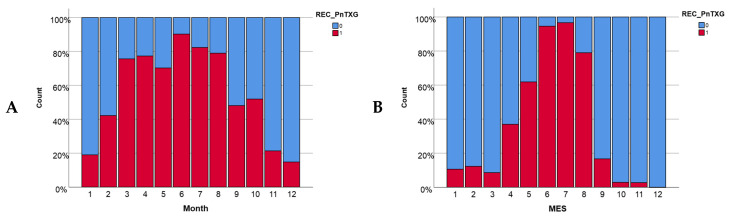
The presence of GYM-A per month (**A**) in Alfacs Bay and (**B**) in Fangar Bay.

The occurrence of PnTX-G is high and varies over time during a year in both bays, with these changes being much more noticeable in Fangar Bay (Figure 3B). In Alfacs Bay (Figure 3A), the presence of PnTX-G was high during all months of the year, presenting its highest values during the spring and summer months. Meanwhile, in Fangar Bay (Figure 3B), high values were also uncovered that indicate a large presence of PnTX-G in the months of May, June, July, and August. The presence of PnTX-G in this bay decreased during the rest of the year to values below 20%, being minimal in October and November and disappearing in December.

**Figure 4:**
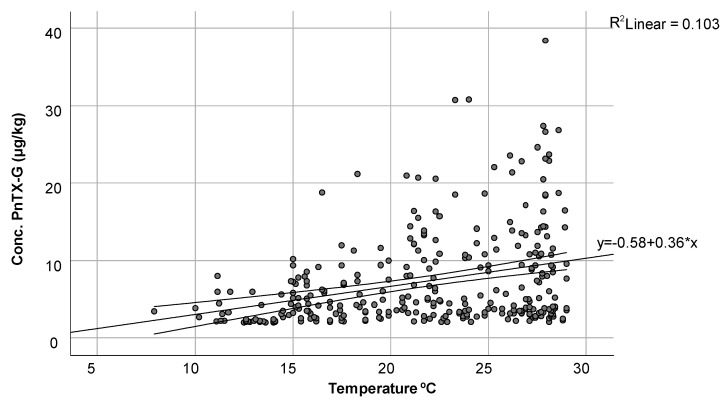
Representation of the variation in the concentration of PnTX-G (µg/kg) with temperature (°C).

For PnTX-G, a more in-depth study regarding its concentrations and temperatures was carried out. Figure 4 represents how the concentration of PnTX-G (µg/kg) varies with temperature, with a linear relationship observed between both variables. As the water temperature increased, so did the concentration of this toxin, reaching high values of 3.65 µg/kg at 20 °C until reaching maximum concentration values of 38.43 µg/kg around 28 °C. The minimum concentration was determined to be 2 µg/kg at 12.5 °C.

The source of PnTXs in the NW Mediterranean coast of Spain has been identified as *Vulcanodinium rugosum* [40]. In our study, it was only detected in one water sample from Alfacs Bay (May 2020) and in three water samples from Fangar Bay (July and August of 2019 and May 2020).

The seawater temperature trends between both bays were very similar. The temperature difference was not extremely significant in this case, coinciding the maximum temperatures with the summer season and the minimum temperatures with the winter season in both bays (Figure S8). Additionally, a study of temperature per year (Figure S9) was carried out. While the maximum temperature of Alfacs Bay (Figure S9A) occurred in the year 2019, the maximum temperature of Fangar Bay (Figure S9B) occurred in the years 2019 and 2020. In this case, it does not seem that there are significant seawater temperature differences between both bays. In 2021, data from the period from January to the beginning of April were represented, so the temperature values decreased by not considering warmer periods.

**Figure 5:**
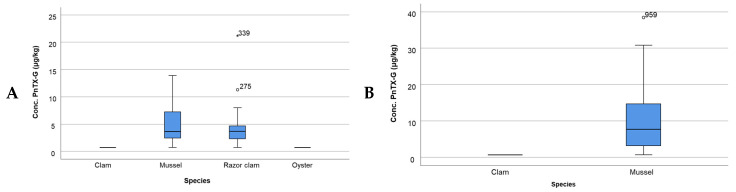
Concentration of PnTX-G by species in (**A**) Alfacs Bay and (**B**) Fangar Bay. Note: dots and asterisks denote outlier observations (atypical data values and extreme data values, respectively.

In the case of PnTX-G, four species of bivalve mollusks were included in this study to examine the variation in the concentration of this toxin among them and between the two bays. In both bays, the maximum concentration values were found in the mussels, with their concentrations being higher in Fangar Bay (68%) (Figure 5B) than in Alfacs Bay (54%) (Figure 5A).

**Figure 6:**
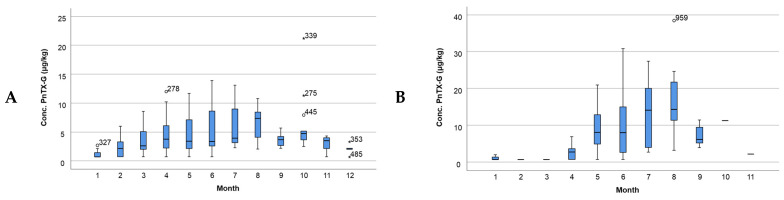
Representation of the PnTX-G concentration by month (**A**) in Alfacs Bay and (**B**) in Fangar Bay. Note: dots and asterisks denote outlier observations.

A temporal evaluation of the concentration of PnTX-G in Alfacs Bay and Fangar Bay over the different months of the year was carried out. Similar trends between both bays were observed, with June being the month when the maximum temperatures were found (Figure 6). In Alfacs Bay (Figure 6A), the concentration of PnTX-G varied throughout the year, being higher in the hottest months, presenting relatively high concentrations of PnTX-G throughout the year. In Fangar Bay (Figure 6B), high concentration values were mainly found in the months from April to September, being minimal the rest of the year.

**Figure 7:**
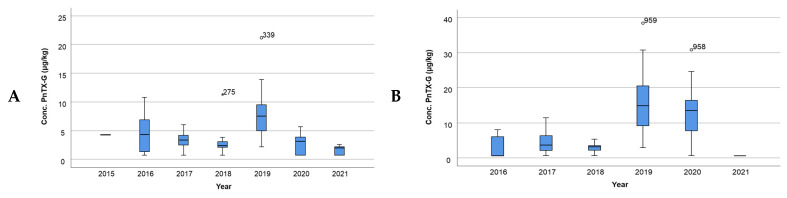
Representation of the PnTX-G concentration per year (**A**) in Alfacs Bay and (**B**) in Fangar Bay. Note: dots and asterisks denote outlier observations.

Furthermore, the variation in the concentration of PnTX-G in each year of the period studied (2015–2021) was evaluated. In both bays, the maximum concentration of PnTX-G occurred in the year 2019 (Figure 7A,B). When this information was studied on a monthly basis, a cyclical trend of the concentration every year was observed. The concentration of PnTX-G was higher during the summer months, decreasing during the winter months for both bays (Figure S10).

## 3. Discussion

This is the first report covering over seven years of CI toxins on the NW Mediterranean coast. Spirolides, gymnodimines, and pinnatoxins comprise a long list of more than 20 analogues [41,42], most of them without the availability of standard reference materials, which complicates the analysis via liquid chromatography coupled with mass spectrometry (LC-MS/MS). Our laboratory has validated this method using alkaline conditions [43] based on Gerssen et al. [42], and has been running analyses since 2013. In recent years, three CIs were incorporated in the lipophilic marine toxin analyses in the routine analyses of the monitoring program following the UNE-EN ISO/IEC 17025:2017 standard [44]. The possible ionic suppression due to the bivalve mollusk matrix was evaluated. A matrix reference material (1.5LQ) was found to be essential within the sequence to correct this matrix effect. Only the representative CI of each group, SPX-1, GYM-A, and PnTX-G, was included in the lipophilic method in order to maintain a good LOQ for all the lipophilic toxins included in this analytical method.

In Catalonia, in the Ebro Delta bays, these three CIs were found at low concentrations (from 26.5 µg/kg to 34.4 µg/kg of SPX-1, <25 µg/kg of GYM-A, and from 2 µg/kg to 38.43 µg/kg of PnTX-G) from January 2015 to April 2021 after the analyses of 1007 samples, of which 36 samples presented trace concentrations of SPX-1 (<25 µg/kg), 13 samples presented traces concentrations of GYM-A (<25 µg/kg), and 144 samples presented trace concentrations of PnTX-G (<2 µg/kg). The quantification limits of SPX-1 and GYM-A were 25 µg/kg, while for PnTX-G, its limit was 2 µg/kg. 

The incidence levels of SPX-1, GYM-A, and PnTX-G in Fangar and Alfacs Bays were variable, presenting higher values in Alfacs Bay. The highest values of SPX-1 (26.52 µg/kg) were recorded in Alfacs Bay, and the highest values of PnTX-G were recorded in Fangar Bay (38.43 µg/kg). For the same area of Catalonia, our values support the results obtained by Garcia-Altares et al. [26], where the confirmation of the first detection of pinnatoxins and spirolides in shellfish samples from Catalonia was described, finding similar values (from 2 µg/kg to 16 µg/kg of SPX-1 and from 2 µg/kg to 60 µg/kg of PnTX-G) after the analyses of 13 samples of mussels and oysters (with only 22 samples having been analyzed).

PnTXs, SPX-1, and GYM-A are widely distributed on the coasts of Spain and Europe. Spirolides, including SPX-1, in mussels (*M. galloprovincialis*) from Galicia (NW Spain) were recorded, presenting low levels ranging from 1.2 µg/kg to 6.9 µg/kg of SPX-1 [35] in 18 samples sampled in May 2015. This study has just been updated with an eight-year study by Blanco et al. [21], describing the 75% of the results below 10 µg/kg with a maximum of 80 µg/kg for SPX-1 from 2014 to 2021 [21]. SPX-1 was also described in Lake Ingril, France (from 1 µg/kg to 19 µg/kg of SPX-1) [29], in the four-year survey on that area and in the EMERGTOX project from 2018 to 2022, where spirolides (SPX-1 and SPX-DesMeD) were systematically present with a maximum concentration of 69 µg/kg [25]. None of the samples that were studied in the previous publications and this study presented concentrations higher than the recommended levels by the toxicology WG of CRLMB 2005 in the EFSA scientific opinion in 2010 [3], where a guidance level of a 400 µg sum of SPXs/kg in shellfish meat was proposed. Additionally, no incidences regarding SPXs have been described.

A study on GYM-A in mollusks from the north and northeast coasts of Spain was carried out by Lamas et al. [33], where the prevalence of this toxin was 6% (122 out of 1900) and the concentrations that were recorded were low with a median of 1.3 to 23.93 µg/kg of GYM-A in mussels. Other countries such as Italy also reported low levels in mussels on the Adriatic coast of Italy (29.2 µg/kg of SPX-1 and 12 µg/kg of GYM-A) [39] and in France with a maximum of 74 µg/kg [25]. Only trace levels of GYM-A were found on the Catalan coast, reaching its maximum values during the winter period

The PnTX-G levels that were uncovered during the study period were low in most cases (ranging from 2 µg/kg to 38.4 µg/kg), which are consistent with other studies that were carried out on the Mediterranean coast of Spain [19,26]. The presence of PnTX-G was also demonstrated in several countries, such as Canada (41 µg/kg) [45] and Norway (150 µg/kg) [36,46], as well as along the Atlantic and Cantabrian coasts of Spain [33], where the levels of PnTX-G on the Galician coasts were quite low (ranging from 2 µg/kg to 15 µg/kg). The highest PnTX-G concentration found in European waters was in Lake Ingril, reaching maximum levels of 1244 µg/kg, and the base levels of this toxin remained above 40 µg/kg [29]. Even though in this study the levels were not as high as the levels detected in Lake Ingril, further evaluations are necessary on the PnTX levels in the NW Mediterranean waters. The levels found in this study (38.4 µg/kg) and the results described by García-Altares et al. [26] (60 µg/kg) are higher than the ones proposed by the French Agency for Food, Environmental and Occupational Health & Safety (ANSES) opinion in 2019 [13], where a regulatory limit of 23 µg/kg was suggested.

In this study, a clear relationship between the presence of toxins and temperature was observed. SPX-1 and GYM-A have a greater presence during the autumn/winter period and then decrease, appearing in trace concentration values during the spring/summer period (Figure 3 and Figure 6). The same seasonal pattern of SPX-1 was observed in Blanco et al. [21], where the highest levels were found during the late autumn–early winter and the lowest at the end of summer [21]. In accordance with Lamas et al. [34], the maximum percentage of GYM-A prevalence was in February and the minimum was in July in Galician waters (Spain) [29]. Otherwise, the appearance of PnTX-G was favored by warm temperatures, showing higher values during the spring/summer period (Figure S10), which is in accordance with Hess et al. [29] and Amzil et al. [25]. These authors found, in their studies that were carried out in Lake Ingril (Mediterranean coast of France), that PnTX-G reached its maximum value (1244 µg/kg) during the summer. On the other hand, the results described by Lamas et al. [33] in Galicia showed a completely opposite trend to the one observed in Mediterranean waters, where PnTX-G appears to especially be prevalent during the winter period on the Atlantic and Cantabrian coasts of Spanish waters.

The presence of *V. rugossum* during the spring and summer periods was in accordance with the seasonal pattern observed in the presence of PnTX-G in bivalve mollusks. This benthic species is rarely detected in the water column; it tends to form temporary benthic cysts on substrates. In the south of France, the absence of this organism from the water column during prolonged periods of shellfish contamination has been observed [29]; when it has been detected in the water column, it is usually of low abundance [47], and non-motile cells are detected on macrophytes. 

## 4. Conclusions

This study has shown the prevalence and concentrations of CIs from the NW Mediterranean coast (Ebro Delta bays) from January 2015 to April 2021. Since 2015, these emerging toxins have been included in the monitoring program and have been analyzed with the LC-MS/MS method for lipophilic toxins in shellfish on a weekly basis. This is the first time that these toxins have been reported for such a long time period.

The effect of seawater temperature was significant for the three toxins, being more important in the case of PnTX-G. The presence of SPX-1 and GYM-A was favored by low temperatures, finding a greater presence during the winter period, while high temperatures favored the increase in PnTX-G, which corresponded to the summer period.

In terms of concentration, SPX-1 was found to a greater extent in oysters, followed by mussels. GYM-A was found in oysters, while PnTX-G was found above all in mussels, followed by razor clams and oysters. The concentration levels that were observed of SPX-1 and GYM-A were higher in Alfacs Bay in comparison with PnTX-G; on the other hand, PnTX-G presented its highest concentrations in Fangar Bay.

CIs should be included in the shellfish safety monitoring programs of lipophilic marine toxins via LC-MS methods, even if they are not regulated, to better assess their presence in shellfish and favor exposure studies that would enable a reliable risk analysis for consumers.

## 5. Material and Methods

### 5.1. Standards and Chemicals

Reference standard solutions were purchased from the Institute for Marine Bioscience of the National Research Council (NRC) from Halifax (NS, Canada), including 13 desmethyl SPX-C (SPX-1, 7 ± 0.4 µg/mL), GYM-A (GYM-A, 5 ± 0.2 µg/mL), and PnTX-G (PnTX-G, 1.92 ± 0.2 µg/mL). Acetonitrile (ACN) hypergrade for LC–MS/MS and MeOH gradient grade for HPLC were purchased from Merck (Darmstadt, Germany). Ammonium hydroxide (25% in water; ≥99.99% trace metal basis) was purchased from Sigma–Aldrich (Steinheim, Germany), and ultrapure water was obtained through a Milli-Q purification system (resistivity > 18 MW cm) from Millipore (Bedford, MA, USA).

### 5.2. Sampling

Different species of bivalve mollusks were collected at various points in the Ebro Delta (NW Mediterranean Sea, Tarragona, Spain), both in Alfacs Bay and in Fangar Bay between January 2015 and April 2021, and they were transported to the Institute of Agrifood Research and Technology (IRTA) laboratories in La Ràpita, Tarragona (Spain). In total, 1007 samples were recollected: 505 samples in Fangar Bay and 502 samples in Alfacs Bay, from the Ebro Delta (Catalonia, Spain).

For the statistical approach, only mussels (*Mytilus galloprovincialis)*, oysters (*Crassostrea gigas)*, and razor clams (*Ensis* sp.) were used, since the CIs were not detected in the other species.

**Figure 8:**
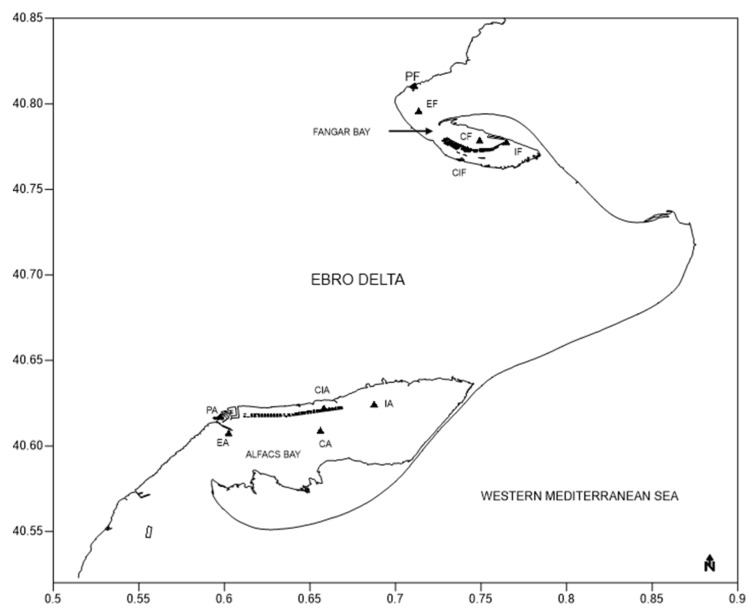
Map of the phytoplankton sampling stations in Alfacs Bay and Fangar Bay, the Ebro Delta.

Water samples for phytoplankton analysis were collected every week at 5 sampling stations in each bay using a silicone hose to obtain a representative sample from the water column (Figure 8). At the central station of each bay, surface and bottom water samples were also collected with weekly sampling frequency. The samples (110 mL) were immediately preserved in Lugol’s solution and transported to the laboratory in a cool box.

At the same sampling stations where the phytoplankton samples were collected, seawater temperature was measured every week using a Professional Plus (Pro Plus) multiparameter instrument from YSI Inc. (Yellow Springs, Ohio, USA).

### 5.3. Water Sample Analysis

Upon their arrival to the laboratory, the samples were acclimatized to room temperature and homogenized, and a 50-mL sub-sample was settled in Utermöhl chambers (HYDRO-BIOS, Altenholz, Germany) for 24 h. After this time, the samples were analyzed under an inverted microscope (Leica DM IL (Leica Microsistemas S.L.U., L’Hospitalet de Llobregat, Spain), following the UNE-EN 15204:2007 standard: Water quality guidance standard on the enumeration of phytoplankton using inverted microscopy (Utermöhl technique) [48].

### 5.4. Shellfish Extraction and Sample Preparation

A triple extraction with methanol was performed on the fully homogenized tissues (1 g) according to the extraction process proposed by Gersen et al. [42], which was also validated by our group [43] based on the EU-Harmonized Standard Operating Procedure for the Determination of Lipophilic marine biotoxins in mollusks via LC-MS/MS (Version 5, January 2015). In summary, 1 g of the homogenized tissue of each sample was weighed in a 15-mL centrifuge tube with a Sartotius 1702 analytical balance, and 3 mL of methanol was added, proceeding to homogenization with a MS2 Minishaker vortex mixer (IKA Labortechnik, Staufen, Germany) for 1 min at 2500 rpm. Subsequently, the samples were centrifuged using a Jouan MR 23i centrifuge (Thermo Fisher Scientific Inc., Waltham, MA, USA) for 5 min at 2000 G and room temperature, and the supernatant was transferred to a 10-mL volumetric matrix. Then, 3 mL of methanol was added to the solid residue in the tubes, and the process was repeated two more times, adding the supernatant to the 10-mL volumetric flask, and a final volume of 10 mL in methanol was prepared (extraction volume ratio 1:10, *v*/*v*). The extracts were filtered through single-use plastic syringes with 0.2-µm PTFE filters into 15-mL centrifuge tubes. For chromatographic analysis, approximately 1 mL of extract was transferred to an amber vial.

### 5.5. Chromatographic Separation

Toxins were separated on a Waters X-BridgeTM C8 column (2.1 mm × 50 mm; 3.5 µm particle size) with a guard column (2.1 mm × 10 mm; 3.5 µm particle size) (Waters, Milford, MA, USA) in an Agilent 1200 LC system (Agilent Technologies, Santa Clara, CA, USA) consisting of a binary pump (G1312B), a four-channel degasser (G1379B), a thermostatted low carry-over autosampler (G1367C + G1330B), and a column oven (G1316B). Alkaline mobile phases were prepared (pH 11) according to Gerssen et al. [49]: mobile phase A consisted of 67 mm ammonium hydroxide with ultrapure MilliQ water, and mobile phase B consisted of 67 mm ammonium hydroxide with 90/10 (*v*/*v*) ACN/MilliQ water. The mobile phases were filtrated through 0.2-µm nylon membrane filters. The column oven temperature was set at 30 °C, and the flow rate was 0.5 mL/min. The elution gradient for the separation was optimized [43] to start with 20% mobile phase B (mpB) and continue with a linear gradient until reaching 100% mpB at 8 min. Then, an isocratic linear gradient of 1 min was maintained at 100% mpB and returned to the initial conditions of 20% mpB in 0.5 min. Finally, the 20% gradient was maintained for 2.5 min before the next injection. The diverter valve was programmed to deliver the eluent from the column to the waste for the first 1.5 min in all of the gradients. The injection volume was optimized at 10 µL, and the sample compartment was set at 4 °C. The outer surface of the needle was flushed with MeOH in the autosampler before every injection.

### 5.6. Mass Spectrometry

A triple quadrupole 3200 QTRAP^®^ mass spectrometer (MS) equipped with a TurboV electrospray ion source (Applied Biosystems, Foster City, CA, USA) operating at atmospheric pressure and in the positive ionization mode (ESI+) was used, employing the following parameters: curtain gas at 20 psi, collision gas at 4 (arbitrary units), ion spray voltage at 5500 V, temperature at 500 °C, nebulizer gas at 50 psi, heater gas at 50 psi, and interface heater ON. The MS was operated in the multiple reaction monitoring (MRM) mode, selecting two product ions per toxin to allow quantification (MRM_1_; the most intense transition) and confirmation (MRM_2_; the confirmation ions for SPX-1, GYM-A, and PnTX-G). Table 1 shows a summary of the MS/MS settings for the cyclic imine analysis. An analytical software “analyst” v1.6 was used for all the MS tuning, the control of the instrument, the acquisition, and the analysis of the data; MultiQuant 3.0.1 software was employed for data treatment.

To perform the calibration curves, external standards were prepared in methanol (LC-MS grade) from an initial 100 ng/mL multi-toxin stock solution of NRC CRM 13-desmethyl SPX-C, NRC CRM PnTX-G, and NRC CRM GYM-A, with 5 dilutions in a range from 2.5 ng/mL to 25 ng/mL for SPX-1 and GYM-A and a range from 0.2 ng/mL to 20 ng/mL for PnTX-G. Samples were injected in duplicate. The sensitivity of the method was evaluated as the slope of the calibration lines and the linearity through two parameters: the correlation coefficients of the quantification line (R^2^ > 0.98) and the deviation of the slopes between consecutive calibration lines (<25%), according to Commission Decision 2002/657/EC [50]. The retention time, together with the width of the peak that is used to obtain the chromatogram of the extracted ions and the signal/noise ratio (S/N), allowed for defining the ratio of the product ions (ion ratio) for each compound. The variation in the retention time values of less than 3% were considered acceptable, as well as the recommendation to maintain a threshold S/N > 3 for the detection limit of the analyte and a threshold S/N > 10 for the quantification limit. In this case, for SPX-1, GYM-A, and PnTX-G, the quantification limits were 25 µg/kg, 25 µg/kg, and 2 µg/kg, respectively.

### 5.7. Statistical Analysis

Statistical calculations were processed using SPSS 26.0, a statistical program typically used for its great ability to work with large databases. The significance tests that were used to evaluate the proportion of the different categories of species and toxins between habits and the different dates were carried out with a G test of independence (a test that was carried out using toxins).

A logistic regression test was also carried out that allows for comparing the complete model with the model of a single constant and Nagelkerke’s R², which is like the ordinary determination of the coefficient [51]. Through this test, the graphs and their corresponding values of temperatures were obtained.

## Figures and Tables

**Table 1:** Transitions monitored, dwell times, decluttering potential (DP), entrance potential (EP), collision cell entrance potential (CEP), and collision energy (CE) of the three CIs that were monitored.

Toxin	Transitions (*m*/*z*)	Time (ms)	DP (V)	EP (V)	CEP (V)	CE (V)	Precursor ion
13-desmethyl Spirolide C (SPX-1)	692.4 > 444.2 692.4 > 426.3	75.0 50.0	96.0 96.0	10.4 7.5	32.0 30.0	49.0 49.0	[M+H]^+^
Gymnodimine-A (GYM-A)	508.3 > 490.4 508.3 > 392.4	75.0 50.0	71.0 71.0	9.0 8.0	26.0 28.0	33.0 45.0	[M+H]^+^
Pinnatoxin-G (PnTX-G)	694.4 > 676.4 694.4 > 164.1	75.0 50.0	111.0 116.0	7.5 8.5	32.0 32.0	43.0 65.0	[M+H]^+^

## Data Availability

All data of the study are in the manuscript.

## Supplementary Materials

The following supporting information can be downloaded at: https://www.mdpi.com/article/10.3390/toxins16010010/s1, Figure S1: Chemical structures of (a) SPX-1, (b) GYM-A, and (c) PnTX-G; Figure S2: Representation of the variation in SPX-1 with temperature; Figure S3: Representation of the presence of SPX-1 by species; Figure S4: Representation of the variation in GYM-A with temperature; Figure S5: Representation of the presence of GYM-A by species; Figure S6: Representation of the variation in PnTX-G with temperature; Figure S7: Representation of the presence of PnTX-G by species; Figure S8: Monthly representation of the average temperature; Figure S9: Annual representation of the variation in the mean temperature; Figure S10: Representation of the variation in the concentration of PnTX-G per year in the study period (from 2016 to 2021).
