# Supplementary material for: The Temporal Distribution of Cyclic Imines in Shellfish in the Bays of Fangar and Alfacs, Northwestern Mediterranean Region
Source: Toxins (Basel). 2023 Dec 23;16(1):10. doi: 10.3390/toxins16010010 (PMC10819045; doi:10.3390/toxins16010010)
Supplement: Supplementary file 1 [file toxins-16-00010-s001.zip › toxins-2727384-supplementary.pdf]

Supplementary Material

Figure Caption

Figure S1. Chemical structures of (a) SPX-1, (b) GYM-A and (c) PnTX-G.

Figure S2. Representation of the variation in SPX-1 with temperature

Figure S3. Representation of the presence of SPX-1 by species

Figure S4. Representation of the variation in GYM A with temperature

Figure S5. Representation of the presence of GYM A by species

Figure S6. Representation of the variation in PnTX-G with temperature

Figure S7. Representation of the presence of PnTX-G by species

Figure S8. Monthly representation of the average temperature

Figure S9. Annual representation of the variation in the mean temperature

Figure S10. Representation of the variation in the concentration of PnTX-G per year in the study period (from 2016 to 2021)

(a)

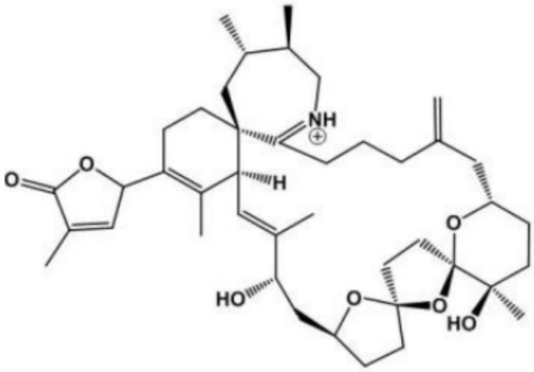

(b)

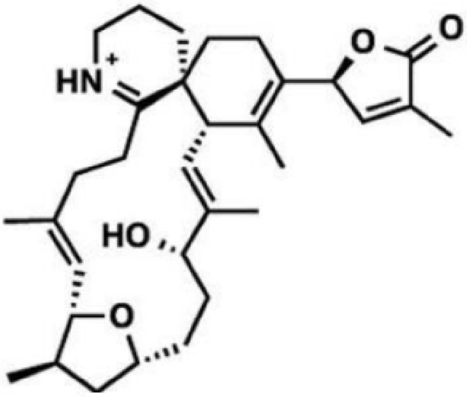

(c)

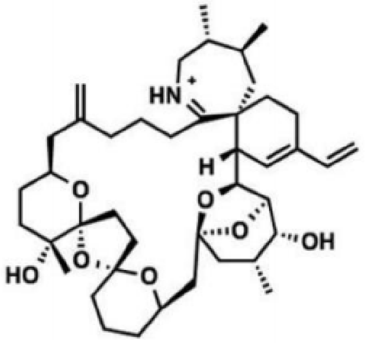

Figure S1. Chemical structures of (a) SPX-1, (b) GYM-A and (c) PnTX-G.

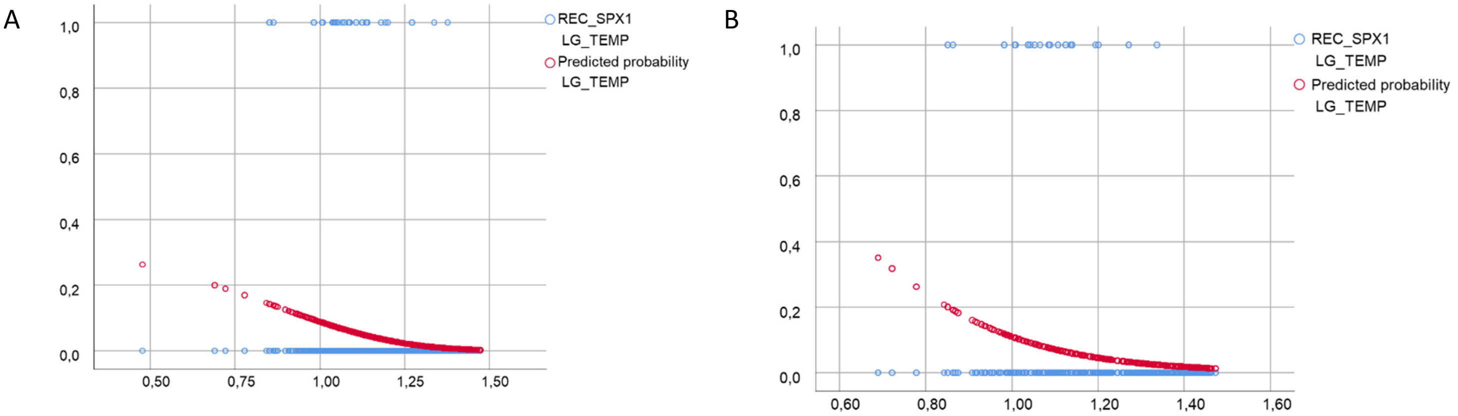

Figure S2. Representation of the variation in SPX-1 with temperature (**A**) in Alfacs Bay and (**B**) in Fangar Bay (both in logarithmic scale, ºC).

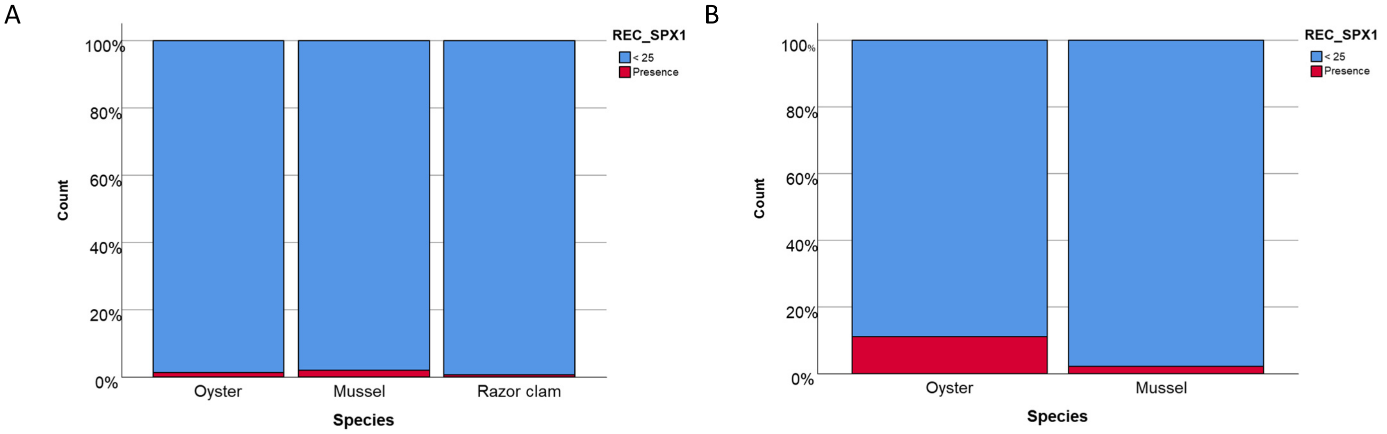

Figure S3. Representation of the presence of SPX-1 by species (**A**) in Alfacs Bay and (**B**) in Fangar Bay. Note: Red: presence and blue: < LOQ.

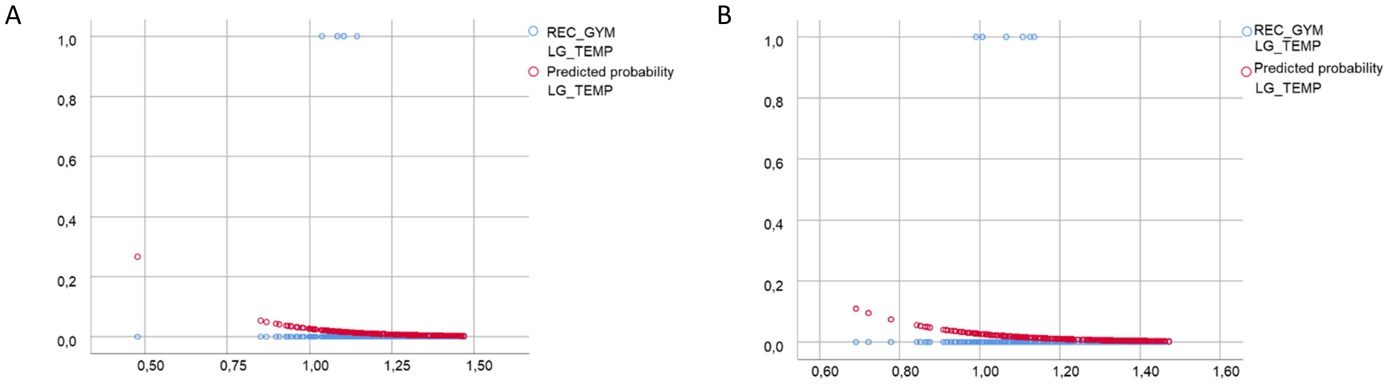

Figure S4. Representation of the variation in GYM A with temperature (**A**) in Alfacs Bay and (**B**) in Fangar Bay (both on logarithmic scale, ºC).

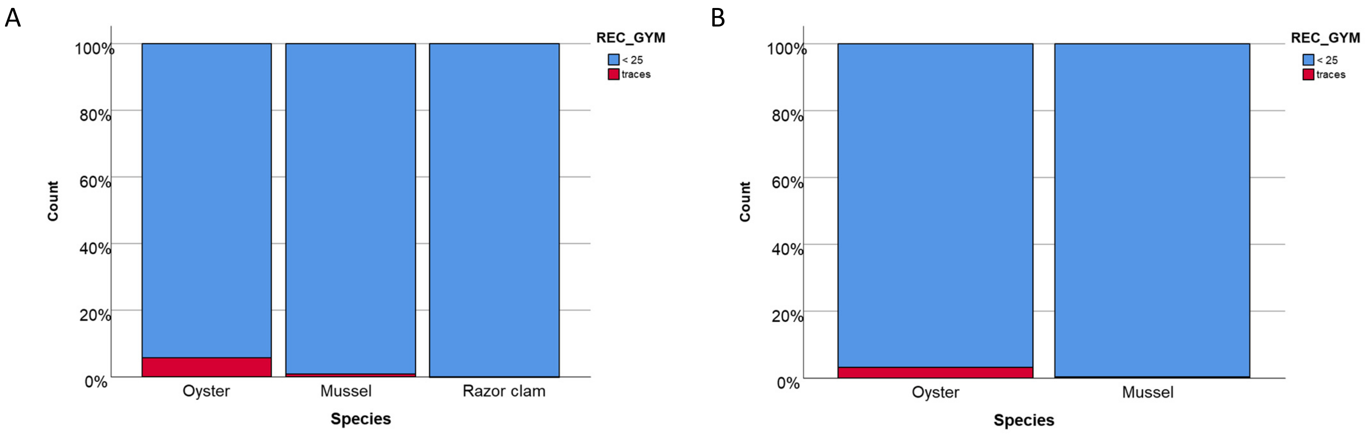

Figure S5. Representation of the presence of GYM A by species (**A**) in Alfacs Bay and (**B**) in Fangar Bay. Note: Red: presence and blue: < LOQ.

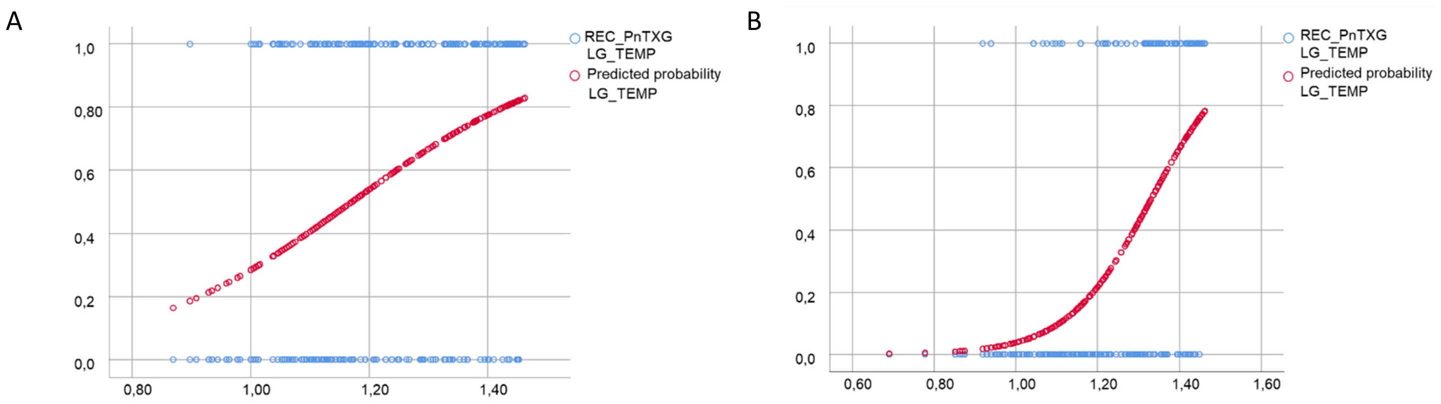

Figure S6. Representation of the variation in PnTX-G with temperature (**A**) in Alfacs Bay and (**B**) in Fangar Bay (both on logarithmic scale, ºC).

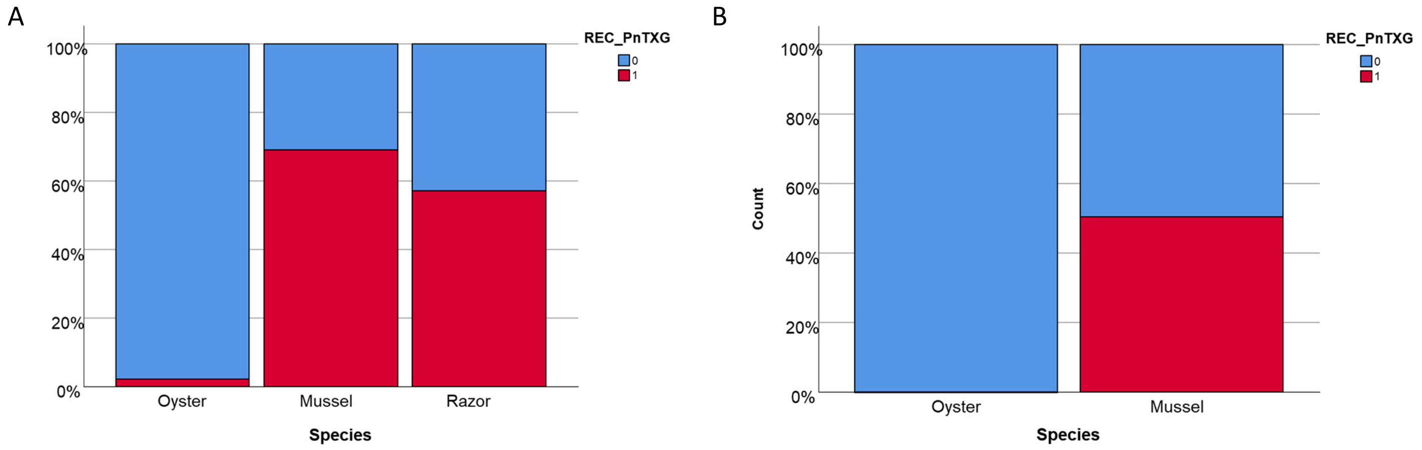

Figure S7. Representation of the presence of PnTX-G by species (**A**) in Alfacs Bay and (**B**) in Fangar Bay. Note: Red: presence and blue: < LOQ.

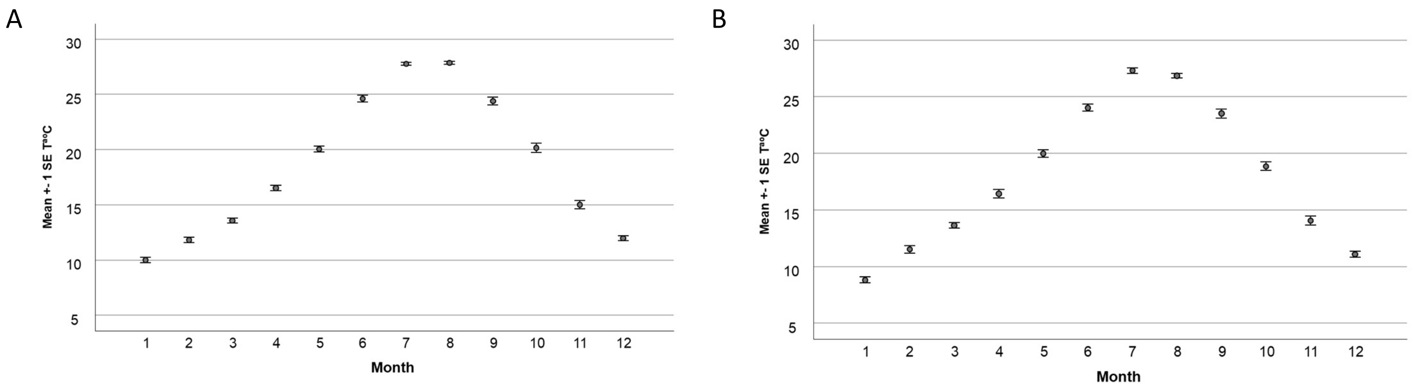

Figure S8. Monthly representation of the average temperature (**A**) in Alfacs Bay and (**B**) in Fangar Bay.

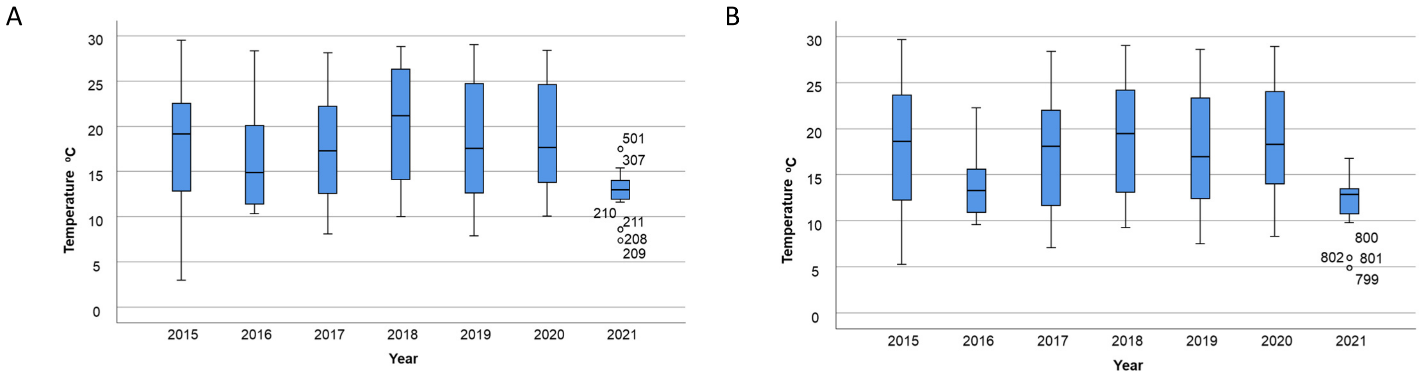

Figure S9: Annual representation of the variation in the mean temperature (**A**) in the Alfacs Bay and (**B**) in the Fangar Bay.

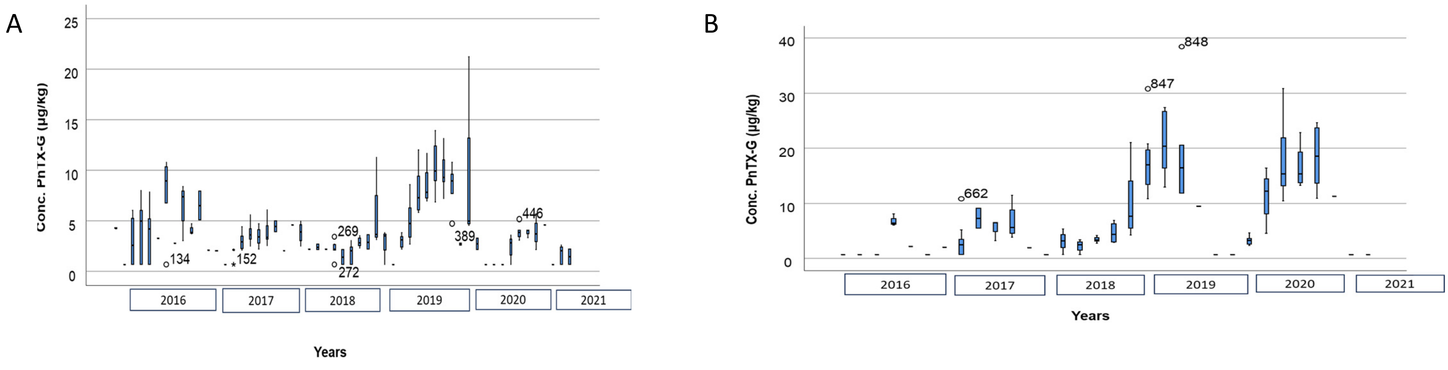

Figure S10: A) Representation of the variation in the concentration of PnTX-G per year in the study period (from 2016 to 2021): (**A**) in Alfacs Bay and (**B**) in Fangar Bay.
